# Work-retirement transitions and mental health: A longitudinal analysis of the role of social protection generosity in 11 countries

**DOI:** 10.1177/14034948211042130

**Published:** 2021-09-11

**Authors:** Ola Sjöberg

**Affiliations:** Swedish Institute for Social Research, Stockholm University, Sweden

**Keywords:** Working conditions, work-retirement transitions, social protection, mental health, comparative

## Abstract

**Aims:**

This study aimed to analyse the effect of work-retirement transitions on post-retirement mental health in individuals with different working conditions in late working life. The focus was on transitions that involve the use of social protection schemes to bridge the gap between the exit from work and retirement, and the extent to which the generosity of such schemes is related to mental health after retirement.

**Methods:**

Individual-level panel data from the Survey of Health, Ageing and Retirement in Europe for 11 European countries were analysed using structural equation models. A total of 1642 individuals who worked in 2004 or 2007 and who retired in 2013 or 2015 were included in the analyses. The outcome measure was mental health as measured by the EURO-D scale.

**Results:**

Respondents with a ‘high strain’ and ‘passive’ work situation have a significantly higher likelihood of using social protection schemes, such as early retirement, sickness, disability and invalidity schemes before retirement. The generosity of such schemes has a significant positive relation to post-retirement mental health.

**Conclusions:**

**This study shows that the generosity of early exit pathways is important for post-retirement mental health, especially for individuals with adverse working conditions at the end of their working lives.**

## Introduction

In light of demographic change and growing old-age dependency ratios, increasing the employment rate of older workers has become a central objective in OECD countries. Policies have been increasingly geared towards extending working life by reducing the generosity of social protection schemes that, in isolation or in combination with paid work, constitute early exit pathways. Such reforms appear to have been successful: the employment rate of workers aged 55–64 across OECD countries increased from 48% in 1990 to around 60% in 2016 [1].

There are important social and individual benefits associated with later retirement. But there are good jobs, and then there are not so good, or even harmful, jobs – jobs that are physically or psychologically demanding, where individuals lack the necessary resources to cope with job-related demands. For workers with such jobs, there is a risk that each additional working year may have negative health consequences not only during working life but also after retirement. Using individual-level panel data from the Survey of Health, Ageing and Retirement in Europe (SHARE) for 11 countries, this study analyses the relationship between early exit pathways from the labour force and mental health after retirement in individuals with different working conditions at the end of their working lives. Previous studies using SHARE suggest that working conditions influence retirement transitions and that the timing of retirement affects post-retirement mental health [2,3]. This study expands on this research by analysing whether the generosity of early exit benefits moderates the relationship between early exits and post-retirement mental health. This study is therefore closely related to research on the relationship between welfare state policies and mental health [4,5].

A large body of literature has shown that adverse working conditions are associated with negative health outcomes. According to the job demand–control model, a combination of high psychological and/or physical job demands and low job control – a ‘high strain’ job situation – is a significant risk factor for developing depressive symptoms and mental ill-health [6–8]. Research also indicates that the effects of unfavourable late-life working conditions are rather persistent in the sense that they can be observed even after retirement [9–11].

Poor working conditions are often seen as a ‘push’ factor, causing employees to exit the labour force early. In such cases, the final effect of late-life working conditions on post-retirement health is likely to depend on the contexts in which these exits take place. One such context is the social protection schemes that are available to individuals if they, partly or completely, leave the labour force before the statutory pension age. In previous research, such schemes have almost exclusively been viewed as a ‘pull’ factor that creates strong financial incentives for, and thereby increasing interest in, early retirement. Rarely have these schemes been analysed in terms of their importance in providing individuals with economic resources during the work-retirement transition process and how these resources may in turn affect post-retirement health. The most relevant strand of research in this context is the body of literature that analyses the association between early retirement and mortality [12]. But very few of these studies have explicitly analysed the importance of income transfer generosity. In studies where this has been analysed, the within-country research design means that the variation in transfer generosity is severely restricted [13,14].

Analysing the relation between early exit pathways, the generosity of these pathways and post-retirement health is difficult for at least two reasons. First, social protection benefits associated with early exit pathways are usually income related. Any within-country differences in benefit generosity associated with early exit pathways at the individual level may therefore reflect variations in earnings capacity. These differences may be contingent upon the health history of individuals, which in turn may be associated with both the likelihood of retiring early and post-retirement health. This makes it difficult to estimate the independent effect of benefit generosity on post-retirement health.

Second, different social protection schemes, such as early retirement, sickness, disability and invalidity schemes, are used in different countries to bridge the gap between early exits from work and entry into the normal old-age pension scheme. Early exit pathways frequently take the form of a sequential combination of different schemes that are often used together with periods of paid work. Therefore, any comparison between nations regarding social protection arrangements and their effect on the workforce exit of older workers that is based on one or a few types of schemes ‘is almost doomed to be misleading’, given the multitude of interrelated work-retirement pathways [15].

The approach taken in this study is to use cross-national variation in early exit benefits when analysing the effect of benefit generosity on post-retirement mental health. It could be argued that this variation is, at least to some extent, exogenous in relation to individual retirement trajectories. Although countries may have used early retirement as a labour-shedding strategy to ease mass unemployment in a similar way, and although governments may react to the citizens’ wishes and expectations in similar ways, it seems reasonable to assume that the variation in policy measures between countries, to some extent, reflects genuine policy alternatives and institutional arrangements [16].

## Methods

### Data and study population

The analyses use longitudinal data from waves 1–6 of the SHARE that were available when this study was initiated. Eleven European countries (AT, BE, CH, CZ, DE, DK, ES, FR, IT, NL, SE) that participated in wave 1 and the so-called SHARELIFE survey in 2008/09 are included in order to obtain as complete information as possible on individual benefit histories.

The SHARE data are based on nationally representative samples of the non-institutionalised population aged ⩾50 years [16,17]. The raw sample used in this study consists of individuals who were aged ⩽62 years and stated ‘working’ as their labour market status in wave 1 (2004) or wave 2 (2007) and were aged ⩾63 years in wave 5 (2013) or wave 6 (2015) and stated ‘retired’ as their labour market status. The sample is thus a cohort of individuals followed over time, and the effective sample size is 1642 individuals. Pre- and post-retirement measures for individuals refer to the first (i.e. wave 1 or 2) and last (i.e. wave 5 or 6) wave, respectively, with non-missing values. Seventy-four per cent of the sample has participated in at least four waves.

### Measures

#### Early exit episodes

Information on individuals’ exit pathways from work is based on the respondents’ benefit and employment history. Respondents were asked if, and for how many months, they had received income from any social protection scheme in the past year and the first year they received it. Waves 2–5 also contain a detailed benefit history, where respondents were asked to indicate the start and stop date (month and year) of each episode when they had received old-age, early retirement, unemployment, sickness and disability benefits. Based on this information, a measure of early exit episodes at the individual level is constructed where the receipt of any form of early exit benefits during a year is given the value 1 (otherwise 0). Since different social protection schemes may perform similar functions in bridging the gap between early exit from work and entry into the normal old-age pension scheme, different schemes that may constitute early exit pathways have been grouped together into one broader category, consisting of early retirement, sickness, disability and invalidity schemes. The relatively small sample size also makes it unfeasible to analyse individual programmes. These data are complemented with data from the employment module SHARELIFE (collected in 2008–2009), which provides retrospective information on sickness periods lasting at least six months for the entire working life of an individual. Two different measures of early exit episodes are used: (a) the proportion of such episodes (in relation to work and old-age retirement episodes) between the ages of 55 and 65 years, and (b) the number of (yearly) episodes with the receipt of benefits from early retirement, sickness, disability and invalidity schemes between the first and last observation (standardised by the number of years between the first and second observation). Two corresponding measures of old-age retirement episodes will also be used as control variables.

#### Generosity of early exit benefits

The measure of the generosity associated with early exit episodes is based on whether the respondent received different social benefits in the past year and how much. Since the amount respondents received through different benefits was not a question asked in the questions covering the detailed benefit history, it is not possible to construct a valid measure of benefits recipiency at the individual level. Therefore, an aggregated country-level measure of the generosity of early exit benefit schemes is used. This measure is constructed by taking the sum that individuals in each country received in early exit benefits (i.e. benefits from early retirement, sickness, disability and invalidity schemes) during a year, divided by the average wage for that country. All respondents who had received these benefits for a whole year across all five SHARE waves were included in the calculation of this measure (*n*=6163). This measure varies between countries from 35% to 65%, with a mean of 48%.

#### Working conditions

Working conditions are operationalised using the well-known demand-control model [18]. Work demands are measured by responses to the following two statements: ‘My job is physically demanding’ and ‘I am under constant time pressure due to a heavy workload’. Job control is measured by responses to the following three statements: ‘I have very little freedom to decide how I do my work’, ‘I have an opportunity to develop new skills’ and ‘I receive adequate support in difficult situations’. Possible responses to the statements are ‘strongly agree’, ‘agree’, ‘disagree’ or ‘strongly disagree’. The job demands and control scales were converted to dichotomous measures according to a median split, and the combination of these two variables led to four work situations: ‘passive’ (low demand, low control), ‘active’ (high demand, high control), ‘high strain’ (high demand, low control) and ‘low strain’ (low demand, high control) as the reference category. Variations of this measure with different categorisations of job situations are also tried.

#### Mental health

Mental health is measured using the EURO-D scale, which is a composite index of 12 items [19]. The maximum score a respondent can get is 12 (‘very depressed’), and the minimum score is 0 (‘not depressed’). A dichotomous measure (0=0; 1⩾0) is also used.

### Confounders

Time-invariant individual-level covariates include birth year, education and sex. Pre-retirement covariates include mental health, job contract, sector, supervisory responsibilities and number of sickness episodes longer than six months up to the first interview. Individual-level covariates measured both pre- and post-retirement include marital status, presence of any chronic disease and hospitalisation in the last 12 months. Post-retirement covariates include old-age retirement episodes (from first interview). Given the small number of countries, only GDP/capita is used as a confounding variable at the country level. Descriptions of variables and descriptive statistics are presented in Supplemental Tables SI and SII.

### Analytical approach

**Figure 1.**
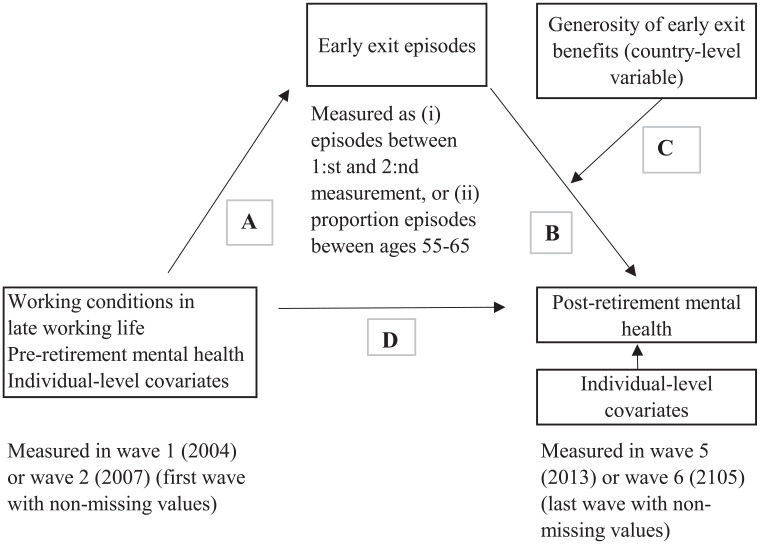
Schematic model of structural equation model.

Since the main explanatory individual-level variables can only be observed before retirement and the number of waves that individuals have participated in varies, the use of traditional panel regression approaches is unfeasible. Instead, a structural equation model approach is used (since all variables are observed, this model may also be called path analysis). A schematic presentation of the model fitted to the data is shown in Figure 1.

Arrow A represents the effect of working conditions (and pre-retirement individual-level covariates) on the likelihood of experiencing early exit episodes during the work-retirement transition. Arrow B represents the effects of such episodes on post-retirement mental health. Arrow C represents whether the generosity of early exit benefits moderates this relationship. Arrow D represents the direct effect of working conditions and other pre-retirement individual-level covariates on post-retirement mental health. Given the small number of countries, this model will be estimated both on each individual country and as two-level random intercept structural equation models for the pooled sample of countries [20]. In the first approach, the coefficients associated with the effect of early exit episodes on post-retirement mental health will be retrieved for each country (i.e. arrow B). The relationship between these coefficients and the generosity of early exit benefits will give a first indication of the extent to which the generosity of such benefits moderates the effect of early exit episodes on post-retirement mental health. In the two-level models, this effect will be estimated directly.

## Results

**Figure 2.**
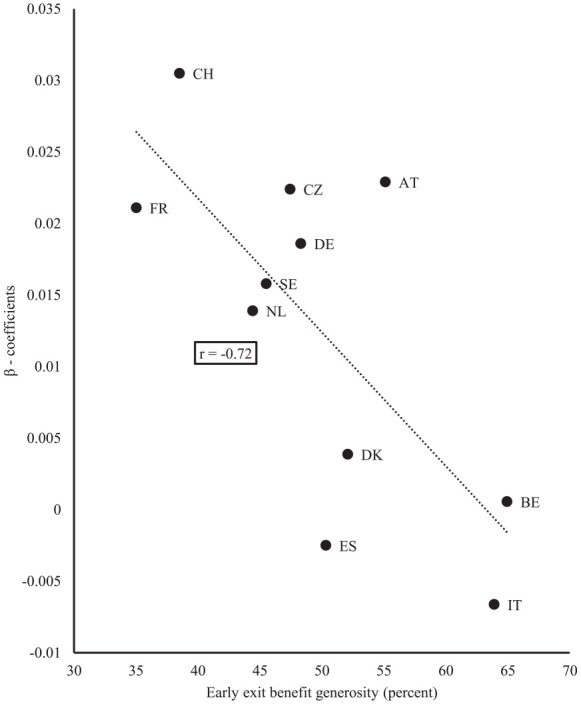
Effect of early exit episodes (proportion episodes between ages 55 and 65) on post-retirement mental health (β coefficients) according to levels of early exit benefit generosity.

Figure 2 indicates that benefit generosity related to early exit episodes moderates the relationship between individuals’ experience of such episodes and post-retirement mental health. In countries with more generous early exit benefits, the effect of early exit episodes (measured as a proportion of such episodes in workers between the ages of 55 and 65) on post-retirement mental health is generally lower than in countries with less generous benefits (*r*=−0.72, *p*=0.013). When early exit episodes are measured as the number of yearly episodes between the first and last interview, this relationship is somewhat weaker (*r*=−0.46). Although this relationship points to the importance if exit benefit generosity, it may also be due to the fact that in countries with more generous benefits, individuals with more moderate health problems at the end of their working lives receive benefits. This could then lead us to overestimate the effect of exit benefit generosity on differences in post-retirement mental health in countries with more generous systems.

Table I presents indirect, direct and total effects on post-retirement mental health for selected variables from two-level random intercept structural equation models. Results for the full set of variables and alternative model specifications can be found in Supplemental Table SIII. Both being in a ‘passive’ and a ‘high strain’ job situation in late working life significantly increases the likelihood of experiencing early exit episodes in relation to the reference category (‘low strain’ jobs). Only a ‘high strain’ work situation has a direct effect on post-retirement mental health, and the total effect is only significant for this form of pre-retirement work situation. Analyses indicate that both high demands and low control are significantly negatively associated with post-retirement mental health. Sickness episodes up to the first observation also significantly increase the likelihood of experiencing early exit episodes. As expected, there is also a significant direct effect of pre-retirement mental health on post-retirement mental health.

**Table I.** Results from structural equation models. Panel A: early exit episodes age 55–65; panel B: early exit episodes between first and second observation. Cluster-robust standard errors.

		Effect on early exit episodes (A)		Total indirect effects (A+B)		Direct effects (D)		Total effects	
*Panel A*									
Working conditions	Passive	2.98	(1.94–4.04)	0.18	(0.11–0.24)	−0.10	(−0.27 to 0.08)	0.08	(−0.10 to 0.26)
(low strain=ref.)	High strain	2.52	(1.29–3.76)	0.15	(0.07–0.23)	0.23	(0.05–0.40)	0.38	(0.15–0.60)
	Active	0.34	(−0.53 to 1.17)	0.02	(−0.03 to 0.07)	−0.00	(−0.09 to 0.08)	0.02	(−0.08 to 0.12)
Mental health 1st observation		−0.18	(−0.41 to 0.04)	−0.01	(−0.02 to 0.01)	0.27	(0.22–0.31)	0.26	(0.21–0.30)
Sickness episodes to 1st observation		6.25	(4.28–8.19)	0.37	(0.24–0.50)			0.37	(0.24–0.49)
Early exit episodes age 55–65 (×100)				5.87	(4.61–7.12)			5.87	(4.61–7.12)
Early exit benefit generosity				0.01	(−0.02 to 0.04)			0.01	(−0.02 to 0.04)
Early exit benefit generosity×sickness episodes age 55–65 (×100) (C)				−0.10	(−0.13 to −0.07)			−0.10	(−0.13 to −0.07)
*Panel B*									
Working conditions	Passive	0.05	(0.03–0.07)	0.15	(0.08–0.23)	−0.10	(−0.28 to 0.08)	0.05	(−0.13 to 0.23)
(low strain=ref.)	High strain	0.04	(0.02–0.07)	0.13	(0.05–0.22)	0.23	(0.05–0.40)	0.36	(0.14–0.58)
	Active	0.00	(−0.01 to 0.01)	0.00	(−0.04 to 0.04)	0.00	(−0.08 to 0.08)	0.00	(−0.09 to 0.10)
Mental health first observation		−0.00	(−0.01 to 0.01)	−0.01	(−0.02 to 0.01)	0.27	(0.22–0.31)	0.27	(0.21–0.30)
Sickness episodes to first observation		0.09	(0.07–0.12)	0.27	(0.19–0.35)			0.27	(0.19–0.35)
Early exit episodes 1st to 2nd observation				2.95	(2.34–3.55)			2.95	(2.34–3.55)
Early exit benefit generosity				0.01	(−0.02 to 0.04)			0.01	(−0.02 to 0.04)
Early exit benefit generosity×sickness episodes 1st to 2nd observation (C) (×100)				−4.60	(−5.79 to −3.39)			−4.60	(−5.79 to −3.39)

Dependent variable: post-retirement mental health (EURO-D). Models adjusted for birth year, education, sex, marital status, presence of any chronic disease, hospitalisation in last 12 months and country-level GDP/capita. Pre-retirement covariates also includes job contract, sector, supervising responsibilities and old-age retirement episodes.

There is also a significant positive effect of early exit episodes on post-retirement mental health, indicating that having experienced such episodes increases the risk of experiencing depressive symptoms after retirement. The significant negative interaction effect between early exit episodes and the generosity of early exit benefits means, however, that the negative effect of early exit episodes on post-retirement mental health is significantly lower in contexts with comparatively generous early exit benefits. Stratified analyses according to sex indicate that the effect of working conditions on the likelihood of experiencing early exit episodes, and the interaction effect between early exit episodes and the generosity of early exit benefits on post-retirement health, are similar for men and women. Stratified analyses according to educational attainment also show similar result over educational groups. The results in Table I are stable in relation to possible influential cases at the country level (Supplemental Table SIV).

**Figure 3.**
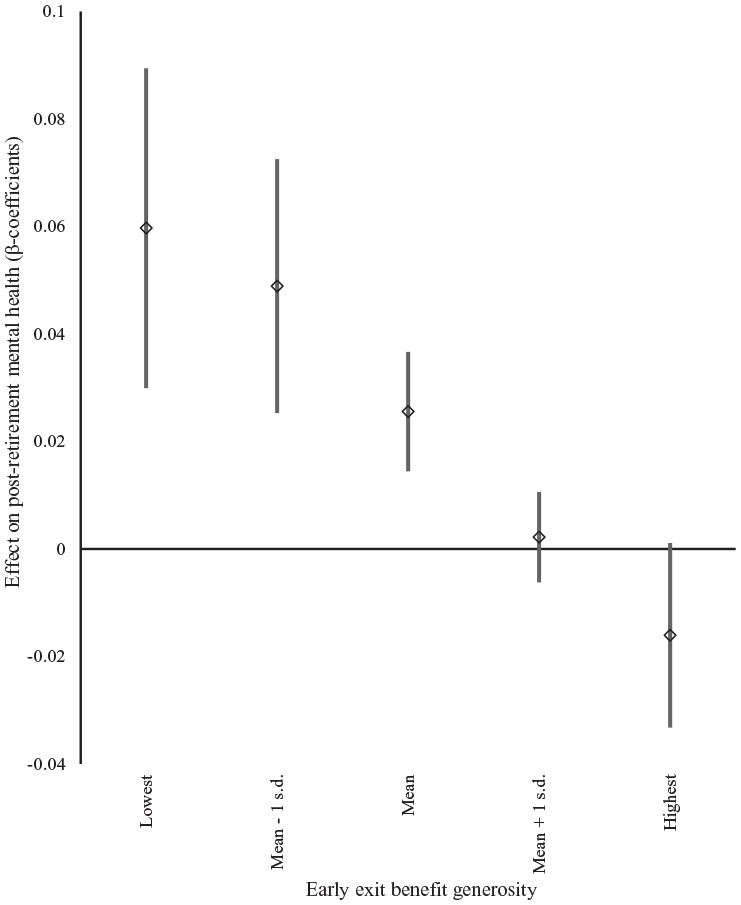
Effect of early exit episodes (proportion) on post-retirement mental health according to levels of early exit benefit generosity for respondents in ‘high strain’ jobs. β coefficients with 90% confidence intervals.

Figure 3 depicts the effect of different levels of early exit benefit generosity (lowest/highest observed values, mean, and mean±1 standard deviation) on post-retirement mental health for individuals with ‘high strain’ jobs. For individuals with ‘high strain’ jobs who experience early exit episodes in countries with above average benefit generosity, there is no significant effect of experiencing such episodes on post-retirement mental health (in relation to the reference category, ‘low strain’ jobs). In contrast, in countries with average or below average generosity, experiencing early exit episodes has a significantly negative effect on post-retirement mental health. These effects are very similar for individuals with ‘passive’ jobs, whereas the generosity of early exit benefits has no significant effect on individuals with ‘active’ jobs. Alternative categorisations of work situations yield very similar results (Supplemental Figure S1).

**Figure 4.**
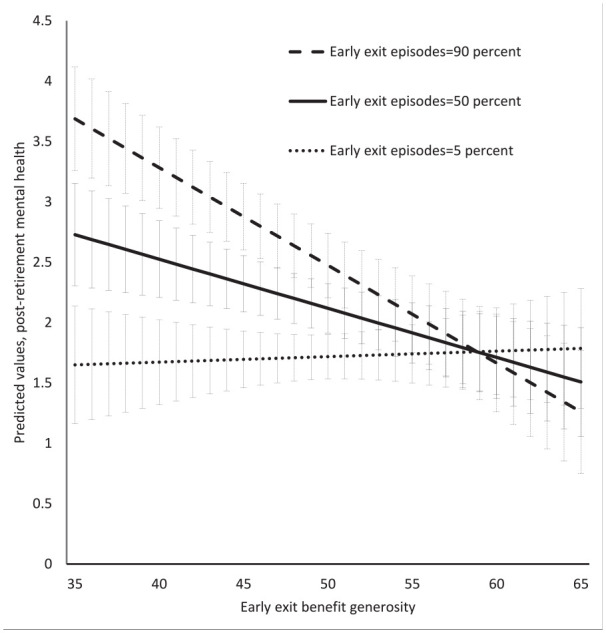
Predicted values for post-retirement mental health for individuals with varying extent of early exit episodes between ages 55 and 65 along values of early exit benefit generosity (bars represent 90% confidence intervals).

Marginal effect analyses also support the finding that the generosity of early exit benefits is positively associated with post-retirement mental health in individuals where early exit episodes constitute an important part of their work-retirement transition. Figure 4 depicts predicted probabilities from the model in panel A of Table I, where early exit episodes constitute 5%, 50% and 90% of total work-retirement episodes along values of early exit benefit generosity (with all other covariates fixed at their mean values).

In individuals for whom early exit episodes constitute a small proportion (5%) of their work-retirement transition, early exit benefit generosity has essentially no influence on their post-retirement mental health. But if early exit episodes constitute a substantial proportion (i.e. 50%) of an individual’s work-retirement transition, early exit benefit generosity has a considerable impact on post-retirement mental health. Comparing individuals where early exit episodes constitute 5% and 50% of their total episodes between the ages of 55 and 65, the 90% confidence intervals do not overlap at early exit benefit generosity above 47% (which corresponds to the mean value of early exit benefit generosity).

## Discussion

This study has demonstrated that individuals with ‘passive’ and high strain’ jobs at the end of their working lives have significantly higher likelihood of using social protection schemes when transitioning from work to retirement. In countries where such schemes are relatively more generous, individuals tend to have better mental health after retirement. This implies that governments and policymakers may be confronted with a trade-off situation that is more complex than previously acknowledged. Earlier research has viewed early exit pathways that enable individuals to leave the labour market – partly or completely – before the statutory retirement age as a powerful ‘pull’ factor that draws individuals towards early retirement. At the societal level, overly generous early exit pathways may therefore lead to budgetary pressure on governments by reducing tax revenue and increasing social expenditure. At the individual level, working later in life may have important social and economic benefits. This study suggests that such effects will have to be weighed against the positive effects of providing relatively generous early exit pathways, especially for individuals with adverse working conditions in late working life.

Several limitations of the present study warrant mention. The data suffered from the usual problems associated with retrospective data collection, such as recall bias, and the comprehensiveness of benefit histories used to calculate early exit episodes varies across individuals. Moreover, it was not possible to calculate a valid measure of early exit benefit generosity at the individual level, and the effect of aggregate benefit generosity is estimated on a small number of countries. With regard to the relation between working conditions and health, the so-called justification hypothesis implies that people who enjoy their work may downplay their health problems and work longer, while those who dislike their work may exaggerate health problems and retire sooner [21]. Furthermore, the sample analysed is a selected group, since many of the individuals facing more serious health problems – possibly caused by harmful working conditions –may have left the labour market before the age of 55 and consequently may not be included in the sample of this study. At the country level, the possibility of labour market exits before the age of 55 is probably related to social protection generosity at older ages, which might mean that individuals with health problems are underrepresented in the samples from countries with more generous exit schemes. It may also be so that also individuals with more moderate health problems at the end of their working lives receive benefits in more generous systems, which could lead us to overestimate the effect of exit benefit generosity on differences in post-retirement mental health in these countries. Finally, it was only possible to include 11 countries in the analyses, and the effective sample size in each participating country is rather small.

Notwithstanding these limitations, this study is the first to analyse the effect of early exit benefit generosity on post-retirement health in a comparative setting. The use of individual-level panel data that allows for the measurement of individual benefit histories and the inclusion of important health-related confounders both pre- and post-retirement are important merits of this study. Overall, the results presented highlight the fact that opportunities to exit the labour force early, either fully or partially, under relatively favourable economic conditions may have important health benefits for individuals with adverse late-life working conditions.

## Conclusions

This study found that the generosity of early exit pathways is important for post-retirement mental health, especially for individuals with adverse working conditions at the end of their working lives. Although reducing the generosity of income transfer schemes may extend working lives, decrease pension expenditure and increase tax revenue, it may do so at the expense of the health of vulnerable individuals with poor late-life working conditions.

## Supplemental Material

sj-xls-1-sjp-10.1177_14034948211042130 – Supplemental material for Work-retirement transitions and mental health: A longitudinal analysis of the role of social protection generosity in 11 countries Click here for additional data file. Supplemental material, sj-xls-1-sjp-10.1177_14034948211042130 for Work-retirement transitions and mental health: A longitudinal analysis of the role of social protection generosity in 11 countries by Ola Sjöberg in Scandinavian Journal of Public Health
